# Human Gait Analysis: A Sequential Framework of Lightweight Deep Learning and Improved Moth-Flame Optimization Algorithm

**DOI:** 10.1155/2022/8238375

**Published:** 2022-07-14

**Authors:** Muhammad Attique Khan, Habiba Arshad, Robertas Damaševičius, Abdullah Alqahtani, Shtwai Alsubai, Adel Binbusayyis, Yunyoung Nam, Byeong-Gwon Kang

**Affiliations:** ^1^Department of Computer Science, HITEC University, Taxila, Pakistan; ^2^Department of Computer Science, University of Wah, Wah Cantt, Pakistan; ^3^Department of Software Engineering, Kaunas University of Technology, Kaunas, Lithuania; ^4^College of Computer Engineering and Sciences, Prince Sattam Bin Abdulaziz University, Al-Kharj, Saudi Arabia; ^5^Department of ICT Convergence, Soonchunhyang University, Asan 31538, Republic of Korea

## Abstract

Human gait recognition has emerged as a branch of biometric identification in the last decade, focusing on individuals based on several characteristics such as movement, time, and clothing. It is also great for video surveillance applications. The main issue with these techniques is the loss of accuracy and time caused by traditional feature extraction and classification. With advances in deep learning for a variety of applications, particularly video surveillance and biometrics, we proposed a lightweight deep learning method for human gait recognition in this work. The proposed method includes sequential steps–pretrained deep models selection of features classification. Two lightweight pretrained models are initially considered and fine-tuned in terms of additional layers and freezing some middle layers. Following that, models were trained using deep transfer learning, and features were engineered on fully connected and average pooling layers. The fusion is performed using discriminant correlation analysis, which is then optimized using an improved moth-flame optimization algorithm. For final classification, the final optimum features are classified using an extreme learning machine (ELM). The experiments were carried out on two publicly available datasets, CASIA B and TUM GAID, and yielded an average accuracy of 91.20 and 98.60%, respectively. When compared to recent state-of-the-art techniques, the proposed method is found to be more accurate.

## 1. Introduction

Person recognition and identification using gait have great importance in the field of machine learning and computer vision [1]. Gait is the walking behavior of a person but to recognize a person by gait from distance and in less illuminated environment it becomes very complicated and difficult [2]. Moreover, as compared to other traditional biometric techniques such as fingerprint, face detection, and iris detection, it does not require direct contact of a person [3]. Due to these discriminative factors, it has taken a lot of attention from researchers and it is used to apply in various applications like security surveillance, dubious person detection, and forensics [4, 5].

In early research, gait recognition was categorized in to two main categories such as model-based and appearance-based [6]. The prior categories are more costly to implement the human model using high-resolution videos and give low average results as compared to modern categories; hence, researchers focus on using modern categories for gait feature detection [7]. In the model-based method, prior information is used to extract the moving features of the human body [8]. Furthermore, in this method, the movement of human body is examined using changing factors like gait path, position of joints, and the torso [9]. This is a challenging method, due to its high computational complications. In the model-free method, gait cycle is used to extract the features from a silhouette, and it is simple to implement due to less computational cost [10].

There are various machine learning and computer vision techniques that are used to overcome the covariate factors like angular deviations [11], lightning conditions [12], and clothing and carrying bags [6, 13], but there exist various challenges in extracting useful features that affect the optimal accuracy results. Feature extraction is considered as the most important step in recognizing gait traits [14], such as if the extracted features are related to the problem, then the system will be able to correctly recognize the human gait patterns. In contrast, if irrelevant features are evaluated, then the system performance will go down and it will not give optimal recognition results [10]. In past, various types of features are used like shape-based features [15], geometrical features [16], and statistical features [17]. Deep features are extracted using deep convolutional neural network techniques to overcome these challenges. Deep learning techniques, rather than manual feature extraction, extract automated features from raw images [18, 19]. In this work, we proposed a sequential lightweight deep learning architecture for human gait recognition. Our major contributions are listed as follows:

- Two pretrained deep learning models are modified namely VGG-19 and MobileNet-V2 based on the target dataset classes and adjusted their weights. Then, both models are trained using transfer learning without freezing any layer and obtained newly trained models.
- Feature engineering is performed on fully connected layer 7 (VGG-19) and global pooling layer (MobileNet-V2) and fused by employing discriminant correlation analysis (DCA)
- A modified moth-flame optimization algorithm is developed for the selection of optimum features that are finally classified using extreme learning machine (ELM)

The rest of the article is organized as follows: Section 2 describes the manuscript's related work.  Section 3 discusses the specifics of selected datasets. The proposed methodology is presented in Section 4.  Section 5 discusses and explains the experimental results. Finally, Section 6 brings the entire manuscript that followed the references to a close.

## 2. Related Works

Identification of human through gait is the most biometric application, and researchers have made extensive studies for it by extracting feature values [20]. In literature, various machine learning and computer vision-based techniques are implemented for human gait recognition [21]. Liao et al. [22] presented Pose-gait model-based gait recognition method. In this approach, the human 3D pose is estimated using CNN, and then spatiotemporal features extracted from the 3D pose are used for the improvement in recognition. Two publicly available datasets CASIA B and CASIA E are used for experimentation, and it gives auspicious results in the presence of covariate factors. Sanjay et al. [23] introduced an automated approach for human gait recognition in the presence of a covariate situation. In the first step, basic distinct stances in the gait cycle are detected which are used to compute the gait features related to these detected stances and it is termed as dynamic gait energy image (DGEI). Then generative adversarial network (GAN) is used to detect the corresponding dynamic gait energy image. These extracted features are then used to compare with the gallery sequences. Finally, GAN-created DGEI is used for final recognition. Publicly available datasets such as CASIA B, TUM Gait, and OU-ISIR TreadMill B are used to validate the presented approach, and it gives considerably improved results as compared to existing methods. Chen et al. [24] introduced a method for cross-view gait recognition using deep learning. Multiview gait generative adversarial network is introduced for creating fake gait data samples for extension in existing data. The method is then used to train each instance of each view involved in single or multiple datasets. Domain alignment using projected maximum mean dependency (PMMD) is utilized to minimize the effect of distribution divergence. CASIA B and OUMVLP are used for experimentation, and the achieved results show that the introduced method gives better results than existing methods. Hou et al. [25] presented a set residual network-based gait recognition model to detect more discriminative features from the silhouettes. Set residual block is introduced to extract the silhouette level and two-level features in a parallel sequence, and then the residual connection is applied to join the two-level features. Moreover, an efficient method is applied to utilize features from the deep layers. Two datasets CASIA B and OUMVLP are used for experimentation. The applied approach gives consistent results as compared to existing methods. Gul et al. [13] introduced a machine vision method to extract the distinct gait features from covariate factors. Spatiotemporal gait features are extracted, and these features are used to train the 3D CNN model to overcome these challenges. Then, the holistic method is used by the model to implement the distinct gait features in the form of gait energy images. Two publicly available datasets OULP and CASIA B are used to test the validity of the introduced method with large gender and age differences. The presented approach gives promising results using CASIA B dataset as compared to existing methods. The methods presented above concentrated on both spatial and temporal data. None of them focused on feature fusion or optimization of extracted features to achieve better results in the shortest amount of time. As a result, in this article, we proposed a lightweight deep learning framework for human gait recognition that not only improves accuracy but also reduces a system's computational time.

## 3. Datasets

### 3.1. TUM GAID Dataset

**Figure 1:**
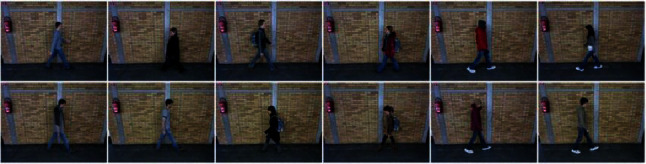
Example images from the TUM GAID gait dataset [26].

TUM Gait from Audio, Image and Depth (GAID) [26] dataset consists of RGB audio, video, and depth. It consists of 305 subjects carried out in two indoor walking sequences in which four distinct situations are captured without any view variation through Microsoft Kinect like six normal walk videos (*n*1–*n*6), two videos with carrying a bag (*b*1–*b*2), and two walking videos wearing coating shoes (*s*1-*s*2), and there is an elapsed time instance in which 32 subjects were recorded wearing distinct cloths. A few sample images are shown in Figure 1.

### 3.2. CASIA B Dataset

**Figure 2:**
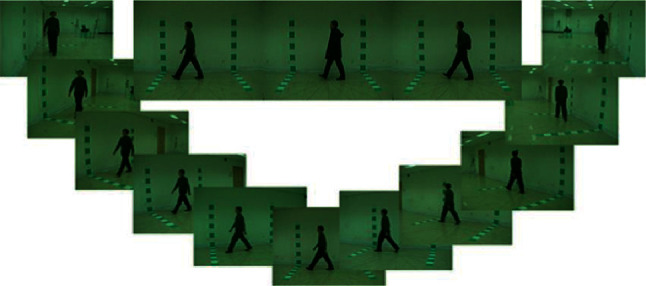
Sample images of the CASIA B gait dataset [27].

CASIA B [27] is a multiview and indoor gait dataset in which 124 subjects are included in recording session of which 93 are male participants and 31 female participants. This dataset considers major factors for gait recognition, that is variation in view angle, clothing, and carrying situations separately. For all, subject videos are captured through a USB camera from 11 different views that include six normal walking videos (NM), two walking videos with wearing a coat (CL), and two walking videos with carrying a bag (BG). A few sample images are shown in Figure 2.

## 4. Methodology

**Figure 3:**
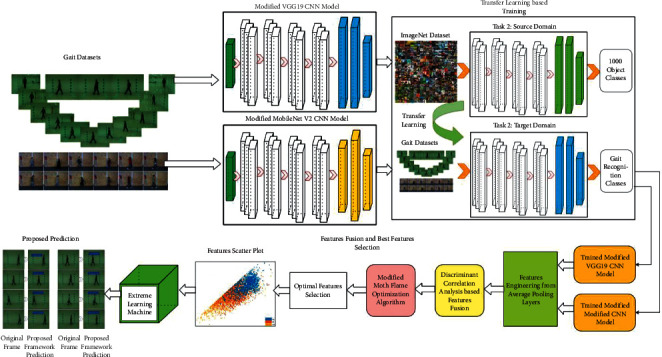
Proposed main flow of human gait recognition using lightweight deep learning architecture.

The proposed lightweight (LW) human gait recognition framework has been presented in this section with detailed mathematical formulations and flow diagrams. The main architecture is shown in Figure 3. In this figure, it is illustrated that the proposed method consists of some important steps such as modification of pretrained CNN models, training of modified models using transfer learning (TL), feature engineering on global average pooling layers, fusion of extracted deep features using discriminant correlation analysis (DCA), selection of best features using the modified moth-flame optimization algorithm, and finally classification using the extreme learning machine (ELM). The details of each step are given.

### 4.1. Convolutional Neural Network (CNN)

The convolutional neural network (CNN) has become an important recognition task in the domain of computer vision. The CNN architecture is employed for feature extraction of an image based on several hidden layers. CNNs have many layers, including convolutional, pooling, fully connected, and others. The convolution layer (CL) is the most important layer of a CNN that performed a 2D convolution process on the input and the kernels through a forward pass. The kernel weights in every CL are assigned randomly and their values are changed at each step by applying the loss function through network training. In the end, the resultant learned kernels may identify some types of shapes within the input images. In CL, three different types of steps are performed like convolution, stack, and nonlinear activation function.

Suppose, we have an input matrix *M* and an output *Z* of the CL, and there are some set of kernels *K*_*l*_, ∀*l* ∈ [1,…, *L*], then the output of the convolution process *O*(*l*) after step 1 is represented as

(1) $$\begin{array}{c} O\left(l\right)=M⊗K_{l},\;\forall l\in \left[1,\ldots ,L\right], \end{array}$$

where ⊗ refers to the convolution process, which is the product of filter and inputs. Second, all *O*(*l*) activation maps are combined to create a novel 3D activation map.

(2) $$\begin{array}{c} R=Q\left(O\left(1\right),\ldots ,O\left(L\right)\right), \end{array}$$

where *Q* represents the combination of operations with channel direction, and *L* is the total number of filters. Third, the 3D activation map *R* is given as input into the activation function and gives the resultant activation map as

(3) $$\begin{array}{c} Z=\text{NLAF}\left(R\right). \end{array}$$

The size *Q* of three main matrices (input, filters, and result) is taken as

(4) $$\begin{array}{c} Q\left(i\right)=\left\{\begin{array}{cc} T_{A}\times U_{A}\times V_{A}, & i=I,\\ T_{P}\times U_{P}\times V_{P}, & i=K_{l},\forall l\in \left[1,\ldots ,L\right],\\ T_{N}\times U_{N}\times V_{N}, & i=Z, \end{array}\right. \end{array}$$

where the variables (*T*, *U*, *V*) represent the size of height, width, and channels of the activation map, and the subscripts *A*, *P*, and *N* represent input, filter, and output, respectively. It contains two equalities. First, *V*_*A*_=*V*_*P*_ refers the channel of input *V*_*A*_ equals to the channels of filter *V*_*P*_. Second, *V*_*N*_=*L* refers the channels of output *V*_*N*_ equals the number of filters *L*. Suppose *Y* represents padding, *S* represents stride, so the result of *T*_*N*_, *U*_*N*_, *V*_*N*_ can be evaluated as

(5) $$\begin{array}{c} T_{N}=1+d_{de}\left[\frac{\left(2\times Y+T_{A}-T_{P}\right)}{S}\right],\\ U_{N}=1+d_{de}\left[\frac{\left(2\times Y+U_{A}-U_{P}\right)}{S}\right], \end{array}$$

where *d*_*de*_ is the floor function. The nonlinear activation ϒ generally selects the rectified linear unit (ReLU) function [28].

(6) $$\begin{array}{c} \gamma_{\text{ReLU}}\left(r_{mn}\right)=\text{ReLU}\left(r_{mn}\right)\\ =\max\left(0,r_{mn}\right), \end{array}$$

where *r*_*mn*_ ∈ *R* is the component of the activation map *R*. At present, ReLU is the mostly used NLAF as compared to the traditional hyperbolic tangent (HT) and sigmoid function (SM) function, that are computed as

(7) $$\begin{array}{c} \gamma_{\text{HT}}\left(r_{mn}\right)=\tanh\left(r_{mn}\right)=\frac{\left(f^{r_{mn}}-f^{-r_{mn}}\right)}{\left(f^{r_{mn}}+f^{-r_{mn}}\right)},\\ \gamma_{\text{SM}}\left(r_{mn}\right)={\left(1+f^{-r_{mn}}\right)}^{-1}. \end{array}$$

### 4.2. Transfer Learning

Transfer learning (TL) is the branch of machine learning that transfer the knowledge of one domain to a different domain within less computational time. Given a source domain *ξ*^*S*^={*F*^*S*^, *P*^*S*^(*ϕ*^*S*^)} with the source task $\lambda^{S}=\left\{{\tilde{F}}^{S},f^{S}\left(\cdot \right)\right\}$ and a target domain *ξ*^*T*^={*F*^*T*^, *P*^*T*^(*ϕ*^*T*^)} with the target task, $\lambda^{T}=\left\{{\tilde{F}}^{T},f^{T}\left(.\right)\right\}$ aims to learn a better mapping function *f*^*T*^(.) for the target task *λ*^*T*^ for the knowledge transfer from the source domain *ξ*^*S*^ and task *λ*^*S*^. Hence, the TL is expressed as follows:

(8) $$\begin{array}{c} \xi^{S}\neq \xi^{T}\text{ or }\lambda^{S}\neq \lambda^{T},\text{ where }\phi =v|v_{i}\in F,\;i=1,2,3,\ldots N. \end{array}$$

**Figure 4:**
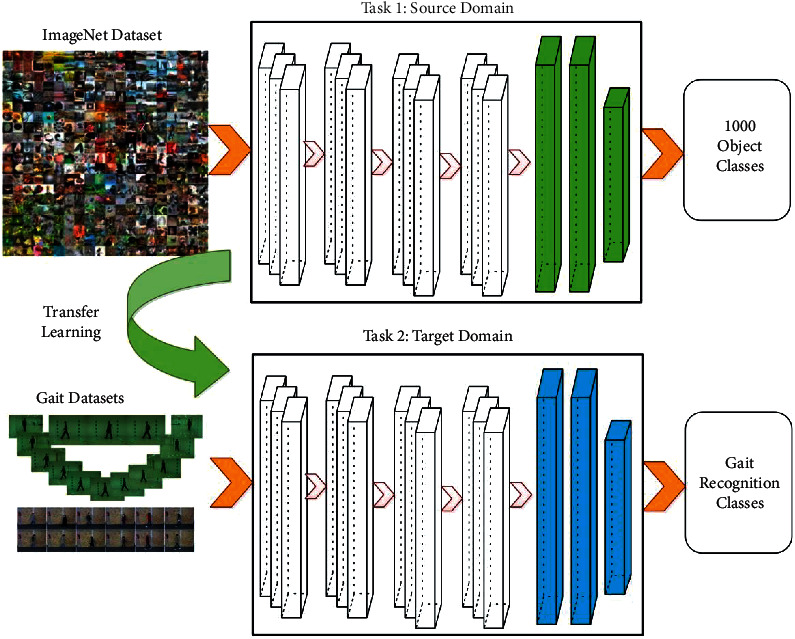
Transfer learning-based training of modified CNN models for human gait recognition.

Hence, DTL is defined as follows: given a TL task $f^{S⟶T}\left(.\right):F^{T}⟶{\tilde{F}}^{T}$ based on [*ξ*^*S*^, *ξ*^*T*^, *λ*^*S*^, *λ*^*T*^], DTL objectives to acquire the *f*^*S*⟶*T*^(.) by leveraging powerful DL process. Visually, the DTL process is shown in Figure 4.

### 4.3. Modified VGG-19 Model

Visual geometry group (VGG)-19 [29] is the modified version of VGG, and it consists of 19 layers with 16 convolutional layers of 64, 128, and 256, and 512 filter sizes with stride length and padding is 1 pixel on each side. The convolutional layer consists of 5 sets, two of them contain 64 filters, the next set contains 2 convolutional layers with 128 filters, the next set contains 4 convolutional layers with 256 filters, and the last two sets contain 4 convolutional layers with 512 filters each. Then, a max pooling layer with a 2 × 2 filter size and stride rate of 2 pixels are used after each set of convolutional layers. The output is then passed to the fully connected layer. Three fully connected (FC) layers and one softmax layer are used for the classification. In this work, we removed the last fully connected (FC) layer and added a new FC layer. Then, several hyperparameters are employed such as training rate, epochs, mini batch size, optimizer, and training loss. Based on these hyperparameters, we trained the modified model from scratch through TL and obtained a new model for only gait recognition task. Later, this modified model is used for the feature engineering task.

### 4.4. Modified MobileNet-V2 Model

MobileNet-V2 [30] is a lightweight CNN-based model specially designed for mobile devices. This architecture can perform well on small datasets as it can overcome the effect of overfitting and it also optimized the memory consumption. In this network, 17 inverted residuals are used between two convolutional layers and one FC layer. So, the depth of the network consists of 53 convolutional layers and one FC layer. The working of this architecture is based on two concepts that include depth-wise separable convolution and the inverted residual methods. In this architecture, a full convolutional layer is replaced with a factorized version that divides the convolution into two separate groups. The first layer is named as depth-wise convolution; its function is to do lightweight filtering by using one convolutional filter on each input channel. The second layer is named as point-wise convolutional layer, which is used for creating new features by computing linear models of the input channels. The depth-wise convolutional layers contains 2 convolutional layers: the first layer contains a 3 × 3 filter size, while the other one has a 1 × 1 filter size. The other two regular convolutional layers have filter sizes of 3 × 3 and 1 × 1. Moreover, in this architecture, ReLU6 is used instead of ReLU as it is more efficient for less accurate computation. Dropout and batch normalization are then applied, where layer activators are standardized to mean zero and unit variance, and then, linear transformation is applied [31].

In this work, we removed the last layer and added a new FC layer. Then, several hyperparameters are employed such as training rate (0.005), epochs (100), mini batch size (32), optimizer (Adam), and training loss. Based on these hyperparameters, we trained the modified model from scratch through TL and obtained a new model for only gait recognition task.

### 4.5. Feature Engineering

Feature engineering is applied on global average pooling layers of both models and obtained two feature vectors of dimension ×*K*_1_ and *N* × *K*_2_. Mathematically, this process is defined as follows:

(9) $$\begin{array}{c} K_{1}=\text{activation}\left(\text{layer}\right),\\ K_{2}=\text{activation}\left(\text{layer}\right), \end{array}$$

where *K*_1_ and *K*_2_ represent the length of feature vectors, and layer defines the selected one like global average pooling. Thereafter, the fusion process is performed using discriminant canonical correlation analysis approach.

### 4.6. Discriminative Canonical Correlation Analysis-Based Fusion

In this work, the DCCA fusion approach is employed for feature fusion. By applying canonical correlation analysis (CCA), the correlated features *l*_*m*_^*v*^*m*_*t*_ and *l*_*n*_^*v*^*n*_*t*_, *t*=1,…, *z*, are extracted and merged for identification. Though the features extracted from related class samples are not utilized, resultantly it becomes the constraint of the recognition capabilities of CCA. Moreover, the basic concept to introduce CCA is for modeling instead of recognition, and correlation *β* refers to the certainty among *l*_*m*_^*v*^*m*_*t*_ and *l*_*n*_^*v*^*n*_*t*_, *t*=1,…, *z*. CCA was more often utilized for modeling and estimation, for instance image extraction and parameter prediction. If the extracted features are for recognition, then the class description of the instances should be utilized to get more discriminatory features. Finally, the class description was fused with the CCA framework for cooperated feature extraction and presented an innovative approach of fused feature extraction for multimodal recognition, termed as discriminative canonical correlation analysis (DCCA). Mathematically, this approach is defined as follows.

Suppose *z* pairs of mean-normalized pairwise instances {(*m*_*t*_, *n*_*t*_)}_*t*=1_^*z*^ ∈ *ξ*^*a*^ × *ξ*^*b*^ access from *p* classes, DCCA can be systematically represented in the below optimization problem:

(10) lmvSlln−μ.lmvShlnlm,lnmax,

(11) $$\begin{array}{c} \text{s.t.}l_{m}^{v}MM^{v}l_{m}=1, \end{array}$$

where the parameters *S*_*l*_ and *S*_*h*_ are used to compute the inside-class association and between-class association, respectively (detailed description is given below), *μ* > 0 adjustable metric that shows the comparative significance of the inside-class association *l*_*m*_^*v*^*S*_*l*_*l*_*n*_ contrasted with the between-class association *l*_*m*_^*v*^*S*_*h*_*l*_*n*_. Moreover, the limitation value represents the scale limitation on *l*_*m*_, *l*_*n*_.

(12) $$\begin{array}{c} M=\left[m_{1}^{\left(1\right)},\ldots ,m_{z_{1}}^{\left(1\right)},\ldots ,m_{1}^{\left(p\right)},\ldots ,m_{z_{c}}^{\left(p\right)}\right],\\ N=\left[n_{1}^{\left(1\right)},\ldots ,n_{z_{1}}^{\left(1\right)},\ldots ,n_{1}^{\left(p\right)},\ldots ,n_{z_{c}}^{\left(p\right)}\right],\\ f_{z_{t}}={\left[\underset{\sum_{k}^{t-1}z_{k}}{\underset{︸}{0,\ldots 0,}}\underset{z_{t}}{\underset{︸}{1,\ldots 1,}}\underset{z-\sum_{k\leq 1}^{t-1}z_{k}}{\underset{︸}{0,\ldots 0}}\right]}^{v}\in Q^{z},\\ 1_{z}\leq {\left[1,\ldots 1\right]}^{V}\in Q^{z}, \end{array}$$

where *m*_*k*_^(*t*)^ refers the *k*th instance in the *t*th class, similarly *n*_*k*_^(*t*)^, and *z*_*t*_ shows the number of instances of *m*_*k*_^(*t*)^ or *n*_*k*_^(*t*)^ in the *t*th class. The matrix *S*_*l*_ is represented as

(13) $$\begin{array}{c} S_{l}=\sum_{t=1}^{p}\sum_{d=1}^{z_{t}}\sum_{y=1}^{z_{t}}m_{d}^{\left(t\right)}n_{y}^{\left(t\right)V}\\ =MGN^{V}, \end{array}$$

(14) $$\begin{array}{c} G=\left[\begin{array}{ccccc} \; & \; & \; & \;\\ \; & \ddots & \; & \; & \;\\ \; & \; & 1_{z_{t}\times z_{t}} & \; & \;\\ \; & \; & \; & \ddots & \;\\ \; & \; & \; & \; & 1_{z_{p}\times z_{p}} \end{array}\right]\in \xi^{z\times z}, \end{array}$$

where *G* represents a proportionate, positively defined, block crosswise matrix, and Matrix (*G*)=*p*. In contrast, the matrix *S*_*h*_ is represented as

(15) $$\begin{array}{c} S_{h}=\sum_{t=1}^{p}\overset{p}{\underset{\begin{array}{c} k=1\\ k\neq t \end{array}}{\sum }}\overset{z_{t}}{\underset{d=1}{\sum }}\sum_{y=1}^{z_{k}}m_{d}^{\left(t\right)}n_{y}^{\left(t\right)V}\\ =\sum_{t=1}^{p}\sum_{k=1}^{p}\sum_{d=1}^{z_{t}}\sum_{y=1}^{z_{k}}m_{d}^{\left(t\right)}n_{y}^{\left(t\right)V}-\sum_{t=1}^{p}\sum_{k=1}^{z_{t}}\sum_{d=1}^{z_{t}}m_{d}^{\left(t\right)}n_{y}^{\left(t\right)V}\\ =\left(M1_{z}\right){\left(N1_{z}\right)}^{V}-{\text{MGN}}^{V}\\ =-{\text{MGN}}^{V}. \end{array}$$

The “=” in the end holds because mean normalization is applied on the instances, hence both *M*1_*z*_=0 and *N*1_*z*_=0 holds. In contrast between equations (13) and (15), the only difference among *S*_*l*_ and *S*_*h*_ is a single negative sign, thus the objective of equation (10) will be (1+*μ*)*l*_*m*_^*v*^*S*_*l*_*l*_*n*_, and this enhancement issue is free from parameter *μ*, consequently *μ* can be excluded. Hence, DCCA can be represented as

(16) lmvMGNVlnlm,lnmax,s.t. lmvMMvlm=1,lnvNNvln=1.

By applying the Lagrangian multiplier technique, it becomes very simple to get main equation of DCCA, which is represented as

(17) $$\begin{array}{c} \left(\begin{array}{cc} - & {\text{MGN}}^{V}\\ {\text{NGM}}^{v} & - \end{array}\right)\left(\begin{array}{c} l_{m}\\ l_{n} \end{array}\right)=\vartheta \left(\begin{array}{cc} MM^{V} & \;\\ \; & YY^{V} \end{array}\right)\left(\begin{array}{c} l_{m}\\ l_{n} \end{array}\right). \end{array}$$

When the vector pairs (*l*_*mt*_, *l*_*nt*_), *i*=1,…, *g*, adjacent to the first *g* largest generalized eigenvalues and attained, let *L*_*m*_=[*l*_*m*1_,…, *l*_*mg*_], *L*_*n*_=[*l*_*n*1_,…, *l*_*ng*_], then both feature extraction and the feature fusion can be performed using FFS-I and II, respectively, where *g* fulfills the limitations *g* ≤ min(*a*, *b*) and *g* ≤ *p*. The formulation returned a fused vector of dimension *N* × *K*_3_, where *K*_3_ ∈ (*K*_1_, *K*_2_). Later on, this resultant vector is further improved using a modified moth-flame optimization algorithm.

### 4.7. Moth-Flame Optimization Algorithm (MFO)

Several nature-inspired optimization algorithms have been introduced in the literature for best feature selection such as genetic algorithm, particle swarm optimization, and moth-flame optimization [32]. The improved moth-flame optimization algorithm is utilized in this work for the best feature selection. Originally, the MFO algorithm was presented by Mirjalili [33]. It is under the populace-based metaheuristics algorithm. In this procedure, first the data flow of MFO begins by randomly generating moths within the resultant space. Then it calculates the positional (i.e., fitness) value of each moth and label the best position by flame. Afterwards, changing the moth place depends on a whole movement function used to attain a better position labeled by a flame. Moreover, it updates the new best positions of the individual. The previous process (i.e., updating of moths' location and generating the new location) until it meets the resultant criteria. The MFO algorithm consists of three major steps that are as follows.

#### 4.7.1. Creating the Initial Population of Moths

As stated in [33], it is supposed that an individual moth can fly in 1D, 2D, 3D, or in hyper-dimensional position. The matrix of moths can be represented as

(18) $$\begin{array}{c} H=\left[\begin{array}{ccccc} h_{1,1} & h_{1,2} & \cdots & \cdots & h_{1,a}\\ h_{2,1} & h_{2,2} & \cdots & \cdots & h_{2,a}\\ \vdots & \vdots & \vdots & \vdots & \vdots \\ h_{m,1} & h_{m,1} & \cdots & \cdots & h_{m,a} \end{array}\right], \end{array}$$

where *m* represents the number of moths' and *a* represents the number of dimensions in the resultant region. Moreover, the fitness values for entire moths' stored in an array are represented as

(19) $$\begin{array}{c} VH=\begin{array}{c} VH_{1}\\ VH_{2}\\ \vdots \\ VH_{m} \end{array}. \end{array}$$

The remaining elements in the algorithm are flames that are represented using D-dimensional space with their fitness/position value function in the following matrix set as

(20) $$\begin{array}{c} P=\left[\begin{array}{ccccc} P_{1,1} & P_{1,2} & \cdots & \cdots & P_{1,a}\\ P_{2,1} & P_{2,2} & \cdots & \cdots & P_{2,a}\\ \vdots & \vdots & \vdots & \vdots & \vdots \\ P_{m,1} & P_{m,1} & \cdots & \cdots & P_{m,a} \end{array}\right],\\ VP=\left[\begin{array}{c} VP_{1}\\ VP_{2}\\ \vdots \\ VP_{m} \end{array}\right]. \end{array}$$

It is important to note that moths and flames both are solutions. The moths are the real search agents that revolve around the search area, while flames are the moth's best position that is obtained yet. Hence, an individual moth hunts around a flame and updates it when it finds the best solution. Following this procedure, a moth never misses its best solution.

#### 4.7.2. Updating Moths' Location/Positions

MFO utilizes three distinct functions to convergent the global optimum of the optimization issues. Mathematically, it is defined as follows:

(21) $$\begin{array}{c} \text{MFO}=\left(L,M,E\right), \end{array}$$

where *L* represents the first random positions of the moths (*πl* : ∅⟶{*H*, *VH*}), *M* represents that the motion of the moths in the search is (*M* : *H*⟶*H*), and *E* represents end of the search process (*E* : *H*⟶true, false). The equation given below represents *L* function, which is used for the implementation of random distribution:

(22) $$\begin{array}{c} H\left(x,y\right)=\left(UB\left(x\right)-LB\left(y\right)\times \text{rand}\left(\right)+LB\left(x\right)\right., \end{array}$$

where *UB* and *LB* refers to the upper and lower bound variables, respectively. As discussed before, the moths fly in the search area by means of transverse direction. There are three conditions that should be followed when applying a logarithmic spiral: (i) The spiral starting point should start from the moth; (ii) the spiral endpoint should be the location of the flame, and (iii) variation in the range of spiral should not extend from the search area. Thus, in the MFO algorithm, the logarithmic spiral can be defined as

(23) $$\begin{array}{c} S\left(H_{x},P_{y}\right)=R_{x}.z^{qb}.\cos\left(2\pi b\right)+P_{y}, \end{array}$$

where *R*_*x*_ represents space between the *x*th moth and *y*th flame (computed by equation 24), *q* represents a solution to define the shape of the logarithmic spiral, *b* represents a random range between [−1, 1].

(24) $$\begin{array}{c} R_{x}=\left|P_{y}-H_{x}\right|. \end{array}$$

In MFO, the equalization among exploitation and examination is affirmed by the spiral motion of the moth near the flame in the search area. Moreover, to escape from falling in the trap of the local goal, the best solution has been kept in each step, and the moths fly around the flames by means of *VP* and *VH* matrices. Then, the update criteria are defined as follows:

#### 4.7.3. Updating the Size of Flames

This part highlights to augment the manipulation of the MFO algorithm (i.e., updating the moths' location in *m* various positions in the search area may minimize the chance of exploitation of the best optimal solutions). However, minimizing the extent of flames helps to overcome this problem using the following equation:

(25) $$\begin{array}{c} \text{FLAME NO}=\text{ROUND}\left(O-c\times \frac{O-c}{I}\right), \end{array}$$

where *O* refers to the maximum number of flames, *c* refers the current number of iterations, and *I* represents the maximum number of iterations. This equation returns the best features; however, during the analysis stage, it is observed that the best selected features contain some redundant information; therefore, we tried to overcome this problem and speedup the selection process based on Newton Raphson (NR) formulation. Mathematically, the NR method is defined as follows:

(26) $$\begin{array}{c} \delta_{n}=\delta_{n-1}-\frac{M\left(\delta_{n-1}\right)}{M^{\prime }\left(\delta_{n-1}\right)}, \end{array}$$

where *δ*_*n*_ ∈ *Sf* and *Sf* represent the selected features of moth-flame. Through the above formulation, a stop value is obtained that added in equation 25 for final selection.

(27) $$\begin{array}{c} \text{FLAME NO}=\text{ROUND}\left(\delta -cf\times \frac{\delta -cf}{I}\right). \end{array}$$

**Figure 5:**
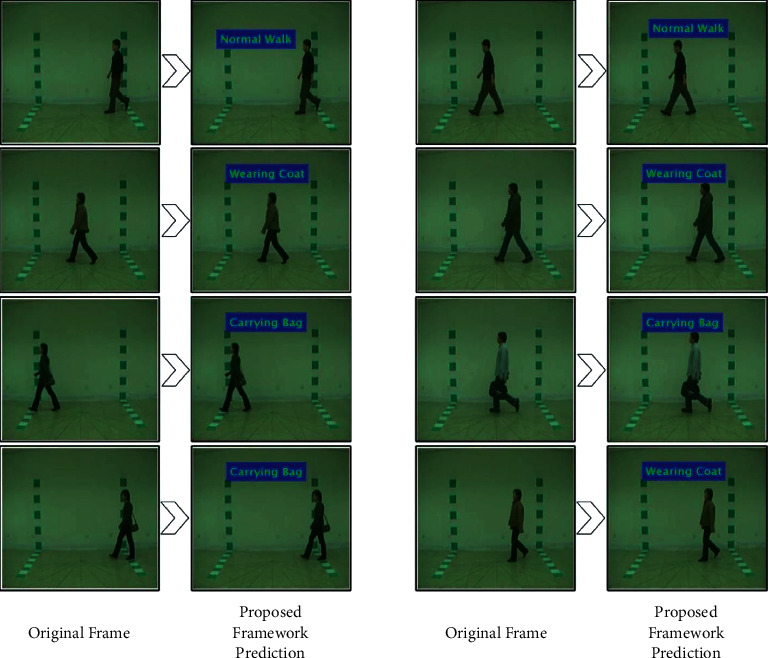
Proposed architecture labeled results on the CASIA B dataset.

The final selected features are passed to the extreme learning machine (ELM) for classification. A few visual predicted frames are shown in Figure 5.

## 5. Experimental Results and Analysis

In this section of the proposed method, the detailed experimental process is presented in the form of tables and graphs. The proposed method is tested using two publicly available datasets, CASIA B and TUM GAID. Section 2 contains more information on both datasets. Instead of 70 : 30, the selected datasets are divided 50 : 50 for training and testing. The main reason for this portioning is to make the validation process more equitable. All of the results are based on 10-fold cross-validation. For the classification results, several classifiers are used, including the extreme learning machine (ELM), support vector machine (SVM), KNN, ensemble tree (EBT), and decision trees (DTs). The entire proposed framework is implemented on MATLAB 2021b using Personal Desktop Computer Corei7, 32 GB RAM, and 8 GB graphics card.

### 5.1. Results

#### 5.1.1. CASIA B Dataset Results

**Figure 6:**
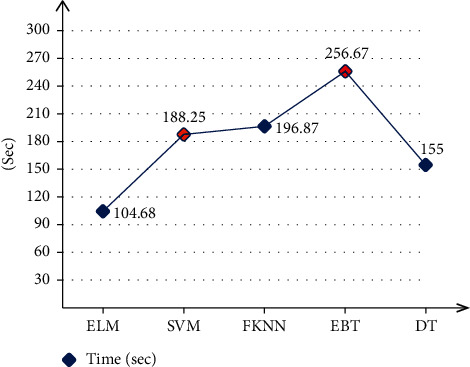
Average classification time of selected classifiers on the CASIA B dataset using the proposed method.

The proposed method results for the CASIA B dataset are presented in the form of numerical values and a time plot in this section.  Table 1 provides the classification results of the CASIA B dataset from all perspectives. Normally, researchers choose only a few angles, but in this work, we chose all 11 angles to test the capability of the proposed algorithm. Each angle has three classes: normal walk (NM), walk with a bag (BG), and walk while wearing a coat (WC) (CL). On this dataset, ELM performed better, with average accuracies of 96.89, 93.07, and 83.66% for NM, BG, and CL, respectively. For each angle, the obtained accuracy is above 90% that shows the proposed method effectiveness. A comparison of ELM with other classifiers such as SVM, FKNN, EBT, and DT shows that ELM performed better than all of them. Moreover, the time is also noted of each classifier as shown in Figure 6. From this figure, it is observed that the ELM and DT classifiers executed fast than the other listed methods.

#### 5.1.2. TUM GAID Dataset Results

**Figure 7:**
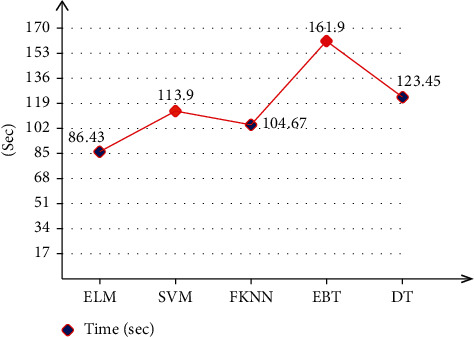
Average classification time of selected classifiers on the TUM GAID dataset using the proposed method.

The results of the proposed method on the TUM GAID dataset are given in Table 2. In this table, accuracy is computed of each class of the selected dataset such as normal walk, walk with a bag, and walk with shoes. Moreover, the average accuracy of each classifier is also computed. Many classifies are selected and ELM shows the better average accuracy of 98.60%. The rest of the classifiers obtained average accuracies of 97.25, 96.73, 96.91, and 96.26%, respectively. The computational time of each classifier is also computed and plotted in Figure 7. It can be seen from this figure that the ELM has a minimum computation time of 86.43 (sec) compared to the rest of the classifiers. Hence, overall, ELM classifier performed better using the proposed method on the TUM GAID dataset.

### 5.2. Discussion and Comparison

A detailed analysis of the proposed framework has been conducted in this section based on confidence interval and standard error means (SEM). As given in Tables 3 and 4, the proposed LightweightDeep-ELM framework gives the better accuracy than other combinations on the CASIA B dataset. Similarly, the proposed framework (LightweightDeep-ELM) also obtained better results on the TUM GAID dataset. Moreover, the average computational time of each classifier for both datasets is also shown in Figures 6 and 7. The ELM execution time is minimum than the rest of the selected classifiers. To further analyze the performance of the ELM classifier, the proposed framework is executed 500 times and computed two values—minimum accuracy and maximum accuracy. Based on the minimum and maximum accuracy, the standard error mean is computed. Through SEM, a confidence error is obtained that shows the consistency of proposed framework.

Table 3 provides the confidence interval-based analysis of the CASIA B dataset. Confidence level and margin of error (MoE) are calculated for each class such as walk, bag, and coat. We selected several confidence levels such as 68.3%, $\sigma_{\bar{x}}$; 90%, 1.645$\sigma_{\bar{x}}$; 95%, 1.960$\sigma_{\bar{x}}$; and 99%, 2.576$\sigma_{\bar{x}}$ and obtained MOE for each is noted as given below. Based on the MoE, it is observed that the proposed framework showed consistent performance on the CASIA B dataset after 500 iterations. Similarly, Table 4 provides the proposed confidence interval-based analysis of the TUM GAID dataset. From this table, it is also confirmed that the proposed method's accuracy is consistent after the numbers of iterations.

At the end, a detailed comparison is conducted with recent techniques for both selected datasets such as CASIA B and TUM GAID.  Table 5 provides the comparison of the proposed method accuracy with recent techniques on the CASIA B dataset. In this table, the authors of [34] obtained an average accuracy of 51.4% on the CASIA B dataset. The authors in [35] improved the average accuracy and reached to 84.2% that was later further improved by [36] of 87.5%. Recently, the authors of [37] obtained an average accuracy of 89.66% on the CASIA B dataset that is improved then the previous noted techniques. Our method achieved an accuracy of 91.20% on the CASIA B dataset that is improved than the existing techniques. Similarly, the comparison of the TUM GAID dataset is given in Table 6. In this table, it is noted that the recently achieved accuracies were 84.4%, 96.7%, 97.9%, and 97.73%. Our proposed method obtained an accuracy of 98.60% that is improved than the recent state-of-the-art (SOTA) techniques.

**Figure 8:**
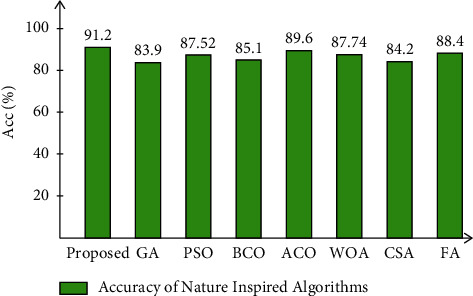
Comparison of proposed optimization accuracy with several other nature-inspired algorithms.

Finally, the improved moth-flame optimization algorithm is compared to several other nature-inspired algorithms (Figure 8) such as the genetic algorithm, particle swarm optimization, bee colony optimization, ant colony optimization, whale optimization, crow search, and firefly algorithm. This graph shows that the proposed optimization algorithm outperforms the other compared algorithms in terms of accuracy. Moreover, the gait is important for several purposes such as assisting those suffering from Parkinson's disease [41, 42]. In this work, we used Adam as an optimizer [18] during the training of deep learning models instead of stochastic gradient descent (SGD). For the gait recognition task, SGD is not performed better than Adam due to a high number of video frames. As we know, Adam is known to be computationally fast, requires less memory, and needs little tuning.

## 6. Conclusion

Human gait recognition using lightweight deep learning models and improved moth-flame optimization algorithm has been presented in this work. Two lightweight pretrained CNN models were fine-tuned and deep transfer learning based trained. Features are extracted from the global average pooling layer and fused using a new approach named DCCA. Furthermore, an optimization algorithm is developed for the selection of the best features. The proposed method was compared based on several classifiers such as ELM and SVM and found ELM is more suitable based on accuracy and time. Two publicly available datasets were employed for the validation process and achieved an improved average accuracy of 91.20 and 98.60%. The key findings of this work are as follows: (i) freezing few middle layers can train a model with less time but it is a chance to sacrifice the better accuracy; (ii) fusion of lightweight models features using DCCA approach is time-consuming but at the end, better information in the form of features is obtained; and (iii) improved optimization algorithm provides the better accuracy and reduces the computational time. In the future, a new scratch-based CNN model will be developed for human gait recognition.

## Figures and Tables

**Table 1:** Proposed classification results of human gait recognition on the CASIA B dataset.

Method	Class	0°	18°	36°	54°	72°	90°	108°	126°	144°	162°	180°	Mean
LightweightDeep-ELM	NM	97.1	98.2	95	93.8	98.1	97.5	98.3	97.2	98	94	98.6	**96.89**
	BG	94.2	95.3	91	92.7	93	89.7	94.8	93.1	92.8	91.8	95.4	**93.07**
	CL	78.8	83.5	82.1	86.3	78.5	90.2	85.9	82	81.3	88.3	83.4	**83.66**

LightweightDeep-SVM	NM	96.2	97.5	96.1	92.8	97.9	96.2	98	97.5	97.1	93.2	98	96.40
	BG	92.5	93.6	93	92.2	94.1	87.6	94	93.5	92.1	92.4	93.4	92.58
	CL	79	82.8	81.5	86	79.1	87.4	83.9	82.3	80.7	87.5	82.1	82.93

LightweightDeep-FKNN	NM	93.2	94.5	92.9	93.1	94.6	91.8	93.5	94.8	94.5	93	94.1	93.63
	BG	87	89.1	90.5	89.6	91.4	82.6	90.8	89.4	87	89.3	90.7	88.85
	CL	73.5	78.4	77.2	81.6	75.3	82.2	80	77.5	76.1	82.4	78.5	78.42

LightweightDeep-EBT	NM	92.8	93.3	90.4	91.5	95	92.6	92.4	93.1	94	93.7	92.9	92.88
	BG	88.5	87.4	90.1	90.5	91.8	82	90.2	90.4	86.1	89.8	91.9	88.97
	CL	72.6	80.1	77	80.4	75	81.6	80.6	76.2	78.5	82	78.1	78.37

LightweightDeep-DT	NM	87.4	88.9	90.1	91.6	93	90.5	88	91.2	91.3	87.4	90.5	89.99
	BG	81.5	82.6	87.5	83.8	90.4	80.6	90	87.9	85.3	84	90.1	85.79
	CL	69.8	72.1	77	78.5	72.7	80	78.3	72.5	72.9	80.1	80.2	75.82

Bold values indicate the best values.

**Table 2:** Proposed classification results of human gait recognition on the TUM GAID dataset.

Classifier	Class-based accuracy (%)			Mean accuracy (%)
	Normal walk	Walk with a bag	Walk with shoes	
LightweightDeep-ELM	**99.64**	**98.52**	**97.65**	**98.60**
LightweightDeep-SVM	98.58	96.92	96.25	97.25
LightweightDeep-FKNN	98.63	96.21	95.37	96.73
LightweightDeep-EBT	98.12	96.82	95.80	96.91
LightweightDeep-DT	97.52	96.03	95.24	96.26

Bold values indicate the best values.

**Table 3:** Confidence interval-based analysis of proposed framework on the CASIA B dataset.

Confidence level	Margin of error
Normal walk	
68.3%, $\sigma_{\bar{x}}$	96.005 ± 0.626 (±0.65%)
90%, 1.645 $\sigma_{\bar{x}}$	96.005 ± 1.029 (±1.07%)
95%, 1.960$\sigma_{\bar{x}}$	96.005 ± 1.227 (±1.28%)
99%, 2.576$\sigma_{\bar{x}}$	96.005 ± 1.612 (±1.68%)

Walk with a bag	
68.3%, $\sigma_{\bar{x}}$	92.59 ± 0.346 (±0.37%)
90%, 1.645$\sigma_{\bar{x}}$	92.59 ± 0.57 (±0.62%)
95%, 1.960$\sigma_{\bar{x}}$	92.59 ± 0.679 (±0.73%)
99%, 2.576$\sigma_{\bar{x}}$	92.59 ± 0.893 (±0.96%)

Walk with a coat	
68.3%, $\sigma_{\bar{x}}$	82.53 ± 0.799 (±0.97%)
90%, 1.645$\sigma_{\bar{x}}$	82.53 ± 1.314 (±1.59%)
95%, 1.960$\sigma_{\bar{x}}$	82.53 ± 1.566 (±1.90%)
99%, 2.576$\sigma_{\bar{x}}$	82.53 ± 2.058 (±2.49%)

**Table 4:** Confidence interval-based analysis of proposed framework on the TUM GAID dataset.

Confidence level	Margin of error
Normal walk	
68.3%, $\sigma_{\bar{x}}$	98.767 ± 0.62 (±0.63%)
90%, 1.645$\sigma_{\bar{x}}$	98.767 ± 1.02 (±1.03%)
95%, 1.960$\sigma_{\bar{x}}$	98.767 ± 1.215 (±1.23%)
99%, 2.576$\sigma_{\bar{x}}$	98.767 ± 1.597 (±1.62%)

Walk with a bag	
68.3%, $\sigma_{\bar{x}}$	97.71 ± 0.573 (±0.59%)
90%, 1.645$\sigma_{\bar{x}}$	97.71 ± 0.942 (±0.96%)
95%, 1.960$\sigma_{\bar{x}}$	97.71 ± 1.123 (±1.15%)
99%, 2.576$\sigma_{\bar{x}}$	97.71 ± 1.475 (±1.51%)

Walk with a coat	
68.3%, $\sigma_{\bar{x}}$	96.925 ± 0.513 (±0.53%)
90%, 1.645$\sigma_{\bar{x}}$	96.925 ± 0.843 (±0.87%)
95%, 1.960$\sigma_{\bar{x}}$	96.925 ± 1.005 (±1.04%)
99%, 2.576$\sigma_{\bar{x}}$	96.925 ± 1.321 (±1.36%)

**Table 5:** Comparison of proposed method results on the CASIA B dataset with recent techniques.

Reference	Year	Datasets	NM	BG	CL	Mean (%)
[34]	2018	CASIA B	68.1	54.7	31.5	51.4
[35]	2019	CASIA B	95.0	87.2	70.4	84.2
[36]	2022	CASIA B	96.0	91.6	74.8	87.5
[37]	2022	CASIA B	96	92	81	89.66
Proposed		CASIA B	**96.89**	**93.07**	**83.66**	**91.20**

Bold values indicate the best values.

**Table 6:** Comparison of proposed method results with recent techniques on TUM GAID.

Reference	Year	Datasets	*N*	*B*	*S*	Mean (%)
[26]	2014	TUM GAID	99.4	59.4	94.5	84.4
[38]	2017	TUM GAID	98.7	91.1	94.5	96.7
[39]	2017	TUM GAID	99.7	98.1	95.8	97.9
[40]	2021	TUM GAID	99.4	97.4	96.4	97.73
Proposed		TUM GAID	**99.64**	**98.52**	**97.65**	**98.60**

Bold values indicate the best values.

## Data Availability

The datasets used in this work are publicly available (http://www.cbsr.ia.ac.cn/english/Gait%20Databases.asp).
